# Interferon lambda in anti-viral defense and cancer: dual roles, mechanism and therapeutic potential

**DOI:** 10.1186/s12967-026-08209-8

**Published:** 2026-05-06

**Authors:** Juliane Blümke, Barbara Seliger

**Affiliations:** 1 Institute of Pathology, Section Immunopathology, Martin-Luther-University Halle-Wittenberg, Magdeburger Str. 2, 06112 Halle (Saale), Germany; 2 Institute of Translational Immunology, Brandenburg Medical School “Theodor Fontane”, Gertrud-Piter-Platz 9, 14770 Brandenburg an der Havel, Germany; 3 Faculty of Health Sciences Brandenburg, Brandenburg Medical School “Theodor Fontane”, Brandenburg, Germany; 4 Fraunhofer Institute for Cell Therapy and Immunology, 04103 Leipzig, Germany

**Keywords:** Type III interferons, Interferon-lambda, Virus infection, Tumor, Immune response, Biomarker, Therapy

## Abstract

**Background:**

The type III interferon family, known as interferon lambda (IFN-λ), consists of four isoforms (IFN-λ1, IFN-λ2, IFN-λ3, and IFN-λ4) and plays essential roles in immune responses. It mediates anti-viral and anti-tumoral activities and is distinguished from type I and type II interferons (IFNs) by its interaction with a unique receptor complex involving IFN-λR1 and IL-10 Rβ predominantly expressed on epithelial cells and selected immune cell populations. This interaction results in activation of the JAK-STAT signaling pathway and transcription of interferon-stimulated genes (ISGs).

**Main body:**

IFN-λs exert anti-viral activities, particularly at epithelial and barrier surfaces, and have emerged as therapeutic agents for chronic viral infections like hepatitis C virus and influenza, providing an alternative to traditional IFN therapies with a more favorable safety profile. Beyond these anti-viral properties, IFN-λs contribute to tumor control by enhancing immune surveillance and modulating the composition of the tumor microenvironment. However, accumulating evidence indicates that IFN-λ cells may also exhibit pro-tumorigenic potential by promoting immune evasion and tumor progression in certain contexts. These opposing functions underscore the complexity of IFN-λ biology and the need for further research to elucidate the mechanisms governing its actions, identify biomarkers that predict IFN response and to develop targeted strategies that maximize its therapeutic benefits, while minimizing adverse effects.

**Conclusion:**

By elucidating the complex interplay between IFN-λ and the immune system, this review provides insights into its dual functions in immune-related diseases, its potential as a biomarker for disease monitoring and prediction of therapy response, and its potential for the development of targeted therapies in cancer treatment and viral infections. However, to improve the patients’ outcomes in infectious diseases and cancer management, a comprehensive understanding of its context-specific effects is required to optimize its clinical application.

Interferon lambda (IFN-λ) has emerged as a multifaceted cytokine with important roles at the interface of innate and adaptive immunity and significant implications in both infectious disease and cancer. As a member of the type III interferon (IFN) family, which includes several closely related proteins, IFN-λ contributes to immune responses against viral infections and tumors. In cancer, IFN-λ exerts anti-tumor effects, but also has the potential to promote tumor growth and immune evasion. Understanding its dual roles is essential for the development of effective therapeutic strategies. This review summarizes the (i) physiological and pathophysiological functions of IFN-λ, (ii) its diverse roles in both anti-viral and anti-tumoral contexts and (iii) it’s potential as a therapeutic agent.

## Characteristics of type III IFNs and their receptors

**Fig. 1:**
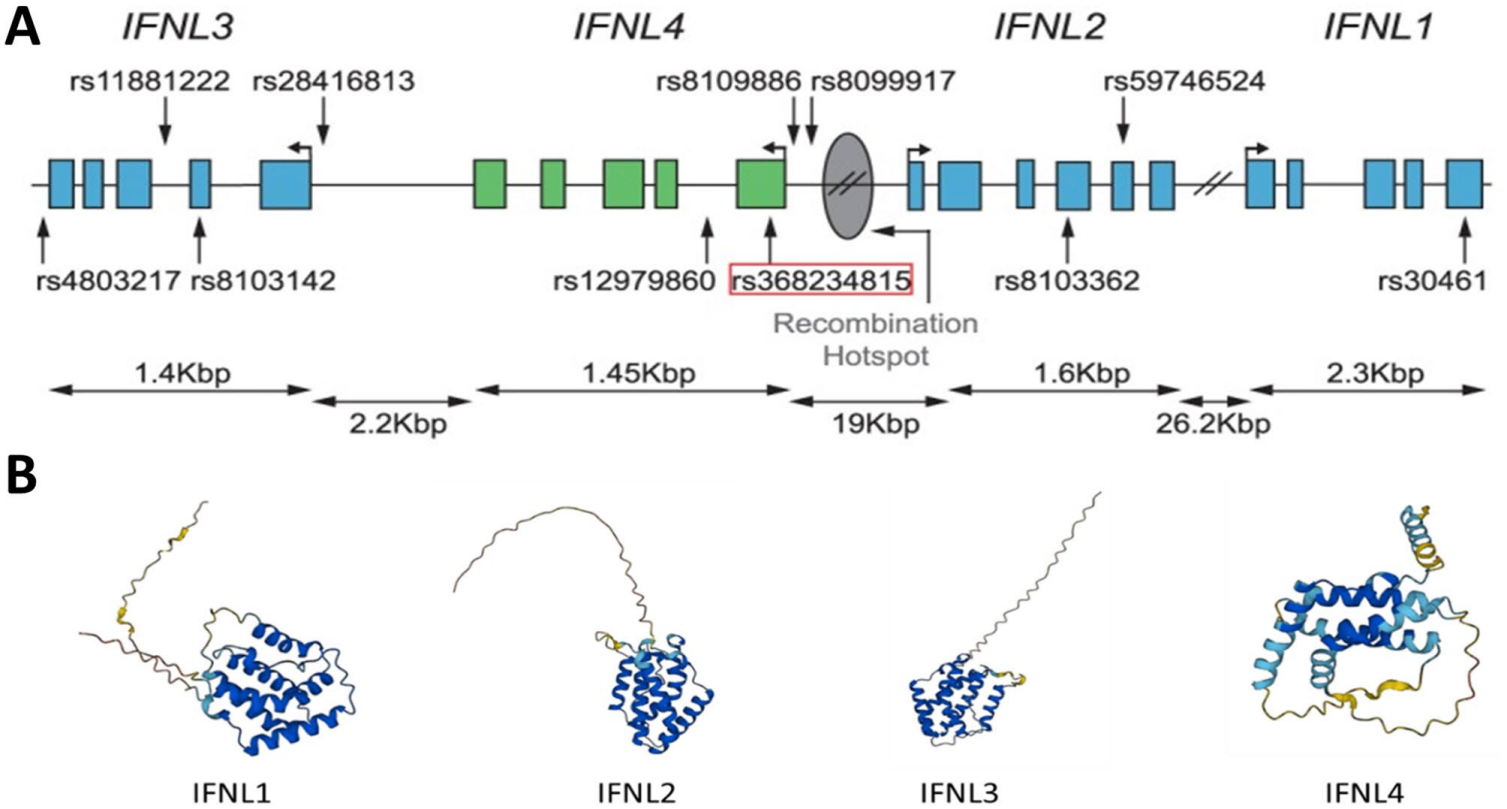
Genomic organization and structural features of IFN-λ family members. **A**. Schematic representation of the chromosomal localization of IFN-λ genes on chromosome 19q13.2 and **B**. the structural relationship among IFN-λ isoforms. IFN-λ1–3 share high sequence homology, whereas IFN-λ4 is more divergent but retains receptor-binding capacity

In general, IFNs are classified into three main types: type I, type II and type III. Type III IFNs, also known as IFN-λ or IFNL, comprise a family of four distinct members named interferon lambda (IFN-λ)1, IFN-λ2, IFN-λ3, also known as IL-29, IL-28A and IL-28B, respectively, and IFN-λ4. All four IFN-λ genes are encoded on chromosome 19q13.2 (Fig. 1A) [1–3].

IFN-λ1, IFN-λ2 and IFN-λ3 proteins exhibit a high amino acid (aa) homology with IFN-λ2 and IFN-λ3 sharing about 96% identity, while IFN-λ1 shares approximately 81% identity with both. IFN-λ4 is more divergent sharing around 30% identity with IFN-λ3, yet retaining similarities in receptor binding regions [2]. In contrast to type I IFNs, type III IFNs possess different exons and maintain a highly conserved structure related to the interleukin-10 (IL-10) cytokine family by adopting a similar four-helix bundle topology [4] (Fig. 1B). However, the sequence homology to IL-10 is relatively low ranging from 11 to 13% aa identity.

The receptors for the IFN-λ family members are highly conserved and characterized by two type III fibronectin domains in the extracellular domain. The functional receptor complex has a ternary structure, where one IFN-λ molecule binds to a heterodimeric receptor complex consisting of IFN-λR1 (also known as IFN-λR1 and IL-28 Rα) and IL-10 Rβ (also known as IL10RB) [1, 3]. This IFN-λ/IFN-λR1/IL-10 Rβ complex exhibits a unique binding topology compared to other cytokine-receptor complexes. The assembly occurs in two steps with initial high affinity binding of IFN-λ to IFN-λR1, which induces a conformational change followed by lower affinity binding of the IFN-λ/IFN-λR1 complex to the IL-10 Rβ subunit [3]. Notably, in contrast to other cytokine families, IL-10 Rβ binds to the distal end of the IFN-λ helical bundle [5, 6]. This receptor system is characterized by a restricted expression predominantly present on epithelial cells and a subset of immune cells thereby confirming selective IFN-λ activity within certain tissues, and cell types [7].

### Expression of IFN-λ and its regulation

In humans, all four IFN-λ family members are expressed, but their expression levels, in particular for IFN-λ4, vary significantly both among individuals and within species. Under physiological conditions, the basal expression is limited and primarily detected in epithelia, but at very low levels. However, their expression is strongly induced in various cell types in response to viral infection or upon stimulation with pathogen-associated molecular patterns, such as poly(I:C) (Table 1 A, B) [8]. The transcriptional regulation of IFN-λ genes is temporally controlled and mainly mediated by NF-$\kappa$B and interferon regulatory factors (IRFs), which bind to specific regions in the IFN-λ promoters. IFN-λ can also be secreted by epithelial cells and various immune cells, but the IFN-λ isoforms produced vary depending on the cell type (Table 1).

**Table 1:** Constitutive and inducible IFN-λ expression

gene	tissue/cell type (human)	basal RNA expression	inducibility	key regulatory features
IFNL1	respiratory epithelium, intestinal epithelium	very low	strongly increased, especially in epithelia	responsive to NF-kB and IRFs [9–11]
IFNL2	respiratory epithelium	very low	strongly increased	similar to IFN-λ1 [9–11]
IFNL3	respiratory/intestinal epithelium, liver	very low	strongly increased	genetic variants [12]
IFNL4	liver (variant-dependent)	very low	increased upon viral stimuli	polymorphism dependent [12]

### IFN-λ-mediated signal transduction and its (patho)physiological function

**Fig. 2:**
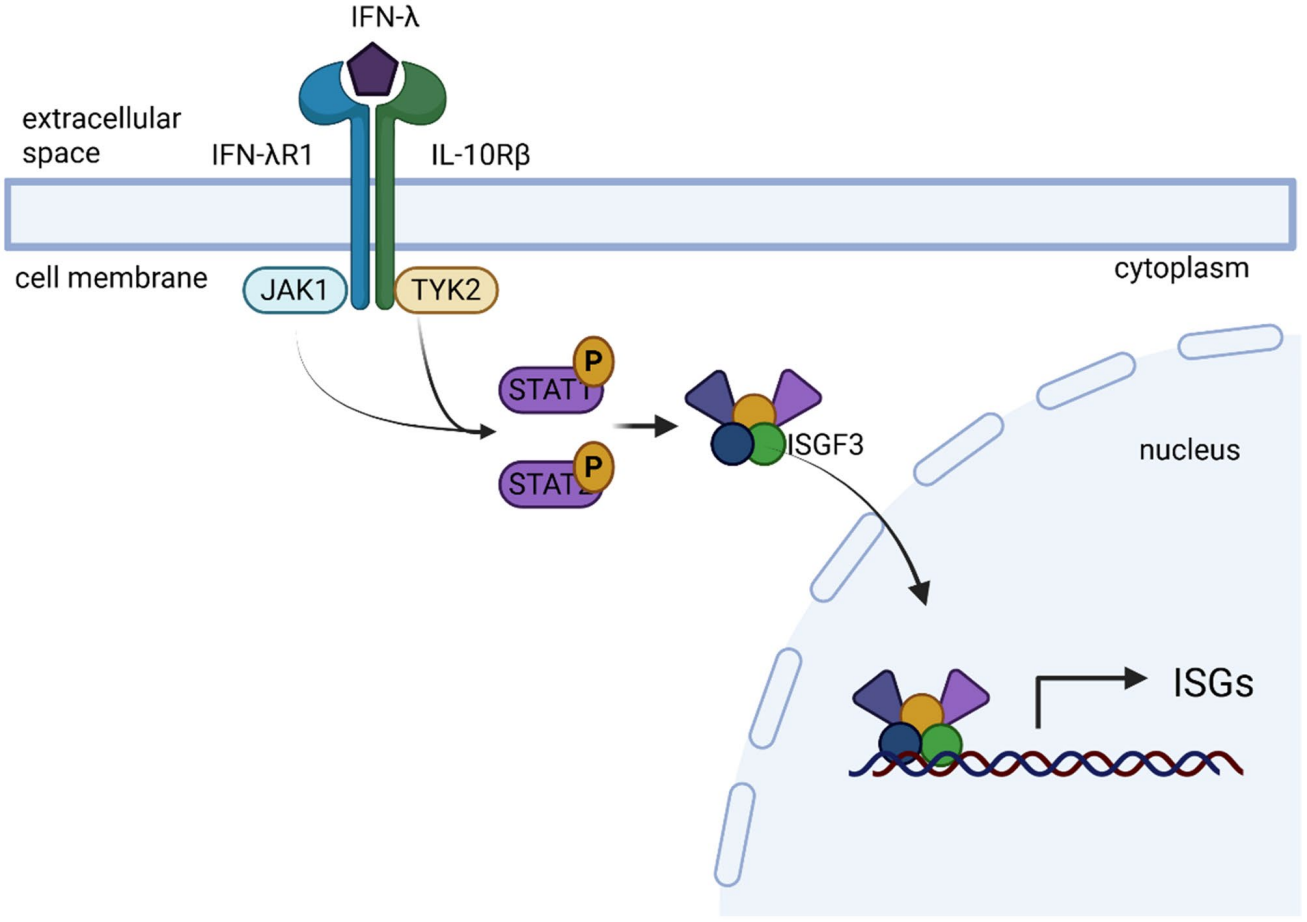
IFN-λ receptor complex and signaling pathway. A schematic diagram of IFN-λ binding to the heterodimeric receptor complex composed of IFN-λR1 and IL-10 Rβ is provided. Ligand binding activates JAK1 and TYK2, leading to phosphorylation of STAT1 and STAT2, the formation of the ISGF3 complex, and finally the transcription of interferon-stimulated genes

Following receptor engagement, IFN-λs activate the Janus kinase (JAK) signal transducer and activator of transcription (STAT) pathway by initiating the phosphorylation of JAK1 and the tyrosine kinase 2 (TYK2), which subsequently phosphorylate STAT1 and STAT2 [13]. These phosphorylated STAT proteins then form a heterodimer, which binds cytoplasmic IRF9, creating the IFN-stimulated gene factor 3 (ISGF3) complex. This complex translocates into the nucleus, where it binds to the interferon-stimulated response elements (ISREs) and induces the transcription of hundreds of interferon-stimulated genes (ISGs) [14]. In addition to this canonical pathway, IFN-λ can activate other STAT proteins, such as STAT3, STAT4 and STAT5, which can either form homo-/ or heterodimers thereby contributing to broader immune responses [15]. In comparison to type I IFNs, the IFN-λ-mediated STAT activation is generally weaker and more sustained. Notably, JAK2, essential for STAT1 phosphorylation, is selectively activated by IFN-λ, which may account for the distinct biological effects observed between IFN-λ and IFN-α/β. Knockout or inhibition of JAK2 specifically disrupts the IFN-λ-dependent signaling cascade without affecting the type I IFN-dependent signaling [16](Fig. 2).

In addition to the STAT1/2 signaling, IFN-λ can activate alternative STAT pathways and interact with the mitogen-activated protein kinase (MAPK) pathway, which contributes to its broader immunomodulatory effects [17–22]. Noteworthy, inhibition of p38 MAPK significantly reduces IFN-λ gene expression, whereas overexpression of p38α MAPK enhances IFN-λ independently of new protein synthesis [23].

### General functions of type III IFNs

Type III IFNs are primarily recognized for their central functions in host–pathogen interactions, particularly their anti-viral defense by inducing ISGs through signaling via their IFN-λR1 and IL-10 Rβ receptor complex on epithelial and certain immune cells. Beyond their anti-viral functions [24, 25], IFN-λ significantly shapes immune cell functions [1, 19], such as macrophage [26–28] and DC polarization, ultimately facilitating T cell activation and proliferation [29]. In addition, IFN-λs promote a T helper (Th)1-biased immune response and influence inflammatory processes, while suppressing Th2 cytokine production thereby supporting cellular and humoral immunity [30]. Furthermore, IFN-λ regulates chemokine expression and immune cell trafficking ensuring efficient pathogen clearance with minimal damage. Moreover, IFN-λ also exert anti-tumoral effects and influence host immune responses by modulating immune cell functions and the tumor microenvironment (TME) [31, 32].

### Similarities and differences between the different types

Although type I (IFN-α, IFN-β) and type II (IFN-γ) IFNs share key signaling pathways with IFN-λ, there exist substantial differences between these IFN families as summarized in Table 2. In contrast to type I and II IFNs, IFN-λs primarily signal through the IFN-λR1/IL-10 Rβ heterodimer [1, 3, 33], expressed on epithelial cells, in particular on those lining the respiratory and gastrointestinal tracts as well as on selected immune cells [1, 3].

**Table 2:** Characteristics of IFN families

features	type I IFN	type II IFN	type III IFN
main members	IFN-α, IFN-β, IFN-ε, IFN-κ, IFN-ω	IFN-γ	IFN-λ1 (IL-29), IFN-λ2 (IL-28A), IFN-λ3 (IL-28B), IFN-λ4
receptor	IFNAR1 and IFNAR2	IFNGR1 and IFNGR2	IFN-λR1 and IL-10R2
structure	0 exon	4 exons	multiple exons (human IFN-λ1/-4 = 5 exons, IFN-λ2/3 = 6 exons
receptor expression	ubiquitous (most cell types) [34]	broad (especially immune cells) [35]	restricted (mainly epithelial cells, some immune cells) [36, 37]
cellular sources	many cells (e.g., DCs, fibroblasts, epithelial cells) [38]	activated T cells, NK cells [34]	epithelial cells, DCs, macrophages [39]
main functions	anti-viral defense, inhibition of viral infection [37, 40] replication, immune modulation	immune regulation, macrophage activation, proinflammatory response, anti-tumoral activity [34]	anti-viral protection at mucosal/epithelial barrier, minor inflammation [36]
signaling pathway	JAK-STAT (ISGF3 complex, ISRE elements) [41, 42]	JAK-STAT (STAT1 homodimers, GAS elements) [42]	JAK-STAT (ISGF3 complex, ISRE elements) [19]
response kinetics	rapid, strong [43]	Moderate [44]	slower, sustained [43]
anti-viral activity	strong, systemic, direct [45, 46]	indirect (via immune activation) [46]	strong, direct, localized [45, 46]
inflammatory potential	Moderate [47–49]	High [35]	Low [50]
anti-tumoral potential	Low [51–53]	High [51, 54]	moderate, context-dependent [55]
clinical use	viral infections, multiple sclerosis [56]	autoimmunity, infections [56]	emerging viral infections [57]

This restricted expression of IFN-λR1 provides a front-line defense against viral infections offering localized activity at mucosal barriers effectively minimizing tissue damage and systemic inflammation [26–28], which is advantageous in preventing immune pathologies associated with excessive inflammation.

Like type I and type II IFNs, type III IFNs also activate the JAK-STAT pathway [58]. Similar to type I IFNs, type III IFNs JAK-STAT activation involves the JAK1 and TYK2 kinases [59–61], while type I IFNs induce rapid and robust anti-viral responses, type III IFNs elicit slower, but more sustained and generally less potent signaling, which is especially prominent at epithelial barriers due to the limited receptor expression [36]. This slower response may be less effective in situations, where immediate anti-viral action is crucial. In contrast, type II IFN predominantly activates immune effector cells, whereas IFN-λ plays a more regulatory role in coordinating immune cell recruitment and epithelial defense. In the context of cancer, IFN-λ has been linked to both tumor development by mediating enhanced inflammatory responses [62], anti-tumoral effects by direct and indirect mechanisms. These include inhibition of tumor cell proliferation, induction of apoptosis and enhancement of antigen presentation. Furthermore, IFN-λ promotes immune cell functions, notably the recruitment of immune cells by shaping the chemokine milieu [63, 64] the activation of NK cells and CD8^+^ T cells thereby strengthening anti-tumor immunity.

To summarize, all three IFN types exhibit key roles in regulating of the immune responses against tumors [65, 66] as well as viral infections [67–69] IFN-λ activities, in particular at the epithelial and mucosal surfaces [70, 71], such as the respiratory [67, 72], gastrointestinal [36] and urogenital tracts as well as the liver [36] Understanding the characteristics of IFN-λ is crucial for developing therapeutic strategies that effectively leverage its advantages, while minimizing its potential drawbacks and possible adverse effects.

### *Role of the* IFN-λ*/IFN-λR axis on immune cells*

Beyond its anti-viral function, IFN-λs significantly influences immune cell behavior [73]. The IFN-λR1 expression on CD4^+^ T cells enables their activation in response to IFN-λ and shapes T cell differentiation by promoting Th1 responses and suppressing Th2 cytokines, such as IL-4 and IL-13, consequently diminishing ongoing Th2 responses [74, 75]. Like IFN-α, IFN-λ produced by DCs in response to TLR stimulation influences their differentiation and maturation [76]. This unique pattern of maturation suggests that while IFN-λ enhances the antigen-presenting capability of DCs, it may not completely activate them, which might have implications for immune responses as characterized by increased expression of MHC class I and II molecules without inducing co-stimulatory molecules [30]. Importantly, IFN-λs counteract the inhibitory effects of transforming growth factor β (TGF-β) or prostaglandin E2 (PGE2) in the TME thereby restoring the IFN-α production and enhancing anti-tumor immunity [77]. IFN-λ-mediated regulation of chemokine expression controls immune cell recruitment and their spatial organization within the tissues. Overall, type III IFNs directly and indirectly engage with a variety of immune cells, mostly promoting immune responses, but also exhibiting immune suppressive functions under certain conditions. These properties enable IFN-λ to coordinate effective immune responses, while minimizing collateral tissue damage.

### Expression of IFN-λ in tumors

There exists evidence that IFN-λ, in particular IFN-λ1, is expressed in tumors of distinct origin either constitutively or in an inducible manner (Table 3). Interestingly, the baseline expression levels of IFN-λ isoforms differ from low to moderate and often correlate with tumor inflammation and prognosis with IFN-λ1 typically downregulated (Table 4). IFN-λ is upregulated in inflamed tumors, typically alongside immune cell infiltration with IFN-λ1 as the most prominent isoform. In bladder cancer, IFN-λ3 expression markedly enhances tumor infiltration of cytotoxic CD8^+^ T cells, Th1 cells, NK cells, proinflammatory macrophages and DCs, while simultaneously decreasing neutrophil infiltration [78]. Transcriptomic analyses revealed a significant upregulation of genes involved in lymphocyte recruitment, phagocytosis aligning with the increased presence of anti-tumor immune cells and altered cytokine secretion associated with an improved sensitivity to anti-PD-1/PD-L1 therapy [78, 79]. In some tumor types, like HNSCC, IFN-λ expression is of prognostic value, particular in inflamed tumors, while elevated IFN-λ1 levels linked to anti-tumor reactivity are found in MSI-high CRC, EBV^+^ B cell lymphoma and inflamed HCC.

**Table 3:** IFN-λ expression across tumor types and immune context

cancer type	baseline expression (primary isoform)	inflamed TME expression	immune context	clinical implication	references
bladder cancer	low (IFN-λ1)	moderate-high (IFN-λ1 > IFN-λ 2/3)	inflamed TME with CD8^+^ T cell infiltration associated with prognostic biomarker and indicator of immune activation	predictive biomarker improved immunotherapy response	[78, 80]
B cell lymphoma	low (IFN-λ 1/3)	moderate-high (IFN-λ 1/ IFN-λ 3)	anti-tumor activity		[81, 82]
breast cancer	low-moderate (IFN-λ 1/2/3)	high (IFN-λ 1/2/3)	enriched in inflamed HER2^+^/TNBC enhanced immune activation [83]	prognostic biomarker (IFNL3)	[83]
colorectal carcinoma	low-moderate (IFN-λ 1/2)	MSI-high inducible (IFN-λ 1)	increased immune activation and apoptosis	potential therapeutic target	[84]
gastric cancer	moderate (IFN-λ 1/3)	high (IFN-λ 1/3)	IFNLR1-high tumor with enhanced IFN-λ signaling	potential biomarker of IFN responsiveness, clinical relevance unclear	[85, 86]
glioma/brain tumors	low (IFN-λ 1)	low-moderate (IFN-λ 1)	limited stromal inflammation; microglia-associated induction	unknown	[87]
head/neck cancer	moderate (IFN-λ 1/2/3)	high (IFN-λ 1/2/3)	HPV^+^-associated immune activation	potential biomarker (virus-associated tumors)	[88–90]
hepatocellular carcinoma	moderate (IFN-λ 1)	high (IFN-λ 1 >>IFN-λ 4)	virus-driven inflammation	prognostic marker and therapeutic target	[91, 92]
leukemia	low (IFN-λ 1/2)	moderate (IFN-λ 1/2/3)	IFN-λ 2/3 upregulation in inflamed bone marrow or viral context	unknown	[59, 93]
lung adenocarcinoma	low-moderate (IFN-λ 1)	high (IFN-λ 1)	inflamed TME in PD-L1^high^ NSCLC; smoker-associated	potential predictive biomarker for immunotherapy response	[94–96]
Melanoma	low (IFN-λ 1/2/3)	moderate-high (IFN-λ 1/2/3)	TIL-rich, inflamed tumors	predictive biomarker for anti-PD1 response	[90]
multiple myeloma	moderate (IFN-λ 1/2)	high (IFN-λ 1)	bone marrow inflammation, IFNLR1-dependent signaling	potential role in disease monitoring, clinical relevance unclear	[97, 98]

**Table 4:** Clinical trials with IFN-λ

Condition	Clinical trial number	Key outcomes
SARS-CoV-2 infection	NCT04354259	accelerated viral clearance
SARS-CoV-2 infection	NCT04331899	reduced viral shedding and symptom duration
HBV infection	NCT02765802	improved viral suppression and decline in HBsAg
HBV infection	NCT03600714	improved viral suppression, enhanced immune activation marker
HBV infection	NCT01204762	anti-viral activity with reduced HBV DNA
HCV (GT 1–4) + HIV infection	NCT01866930	combination efficacy with acceptable safety and anti-viral activity
HCV infection GT 2 or 3	NCT01616524	viral response efficacy-dependent on genotype and treatment regimen
HCV infection GT1	NCT01598090	enhanced treatment responses
Hemophilia + HCV infection GT 1–4	NCT0174154	safe and effective viral suppression
HCV infection	NCT01001754	anti-viral activity with improved safety profile

### Role of IFN-λs in tumor biology

An important function of IFN-λ members is their influence on both innate and adaptive immunity [29, 62] contributing to inhibition of tumor growth through cytokine production [99] and enhanced antigen presentation [33, 100, 101], collectively supporting anti-tumoral functions [59, 60, 65] direct and indirect effects in various preclinical models [78, 102]. In tumor cells expressing the IFN-λ receptor, IFN-λ activates the JAK/STAT pathway leading to the induction of ISGs and anti-proliferative programs. Key effects include inhibition of tumor cell proliferation, induction of cell cycle arrest (e.g., via p18 and p27 upregulation), promotion of apoptosis and induction of DNA damage responses. It is noteworthy that IFN-λ1 has been reported to induce stronger anti-proliferative and DNA damage activities than IFN-λ2 or IFN-λ3 in some models. This is in line with reports in different murine models of melanoma and colon cancer [59, 90].

However, these anti-tumoral effects of IFN-λ are highly dependent on the expression of the IFN-λR1, which is often downregulated in many malignant cells when compared to benign counterparts [36, 103]. Although systemic NK cell activation is limited [102], IFN-λ promotes local recruitment of NK cells to the TME. Furthermore, combination of IFN-α and IFN-λ acts synergistically within the TME to enhance immune-mediated targeting of tumor cells [102].

To sum up, IFN-λ exerts its anti-cancer effects through direct mechanisms that induce antiproliferative and pro-apoptotic responses in tumor cells as well as indirect mechanisms by promoting the recruitment and activation of effector cells into the TME. Understanding these dynamics further emphasizes the potential of IFN-λ as therapeutic agent in cancer treatment, particularly in enhancing the efficacy of immunotherapies.

### IFN-λ *in cancer therapy*

IFN-λ has attracted considerable attention as a potential cancer therapeutic due to its restricted receptor expression, which limits systemic toxicity. It exerts direct anti-tumor effects across multiple tumor types including melanoma, colorectal, lung, breast, prostate cancers and hepatocellular carcinoma by inhibiting proliferation, inducing cell cycle arrest, and promoting apoptosis [104]. In murine melanoma models, IFN-λ delays tumor growth and enhances natural killer (NK) cell infiltration and cytotoxicity thereby contributing to the modulation of the TME. Moreover, IFN-λ improves chemotherapy efficacy when used in combination regimens [102]. In studies involving hepatoma mouse models, a combination therapy of IFN-λ and IFN-α demonstrated an improved tumor eradication and immunity compared to monotherapy, while mitigating IFN-α-associated toxicities [102]. Beyond its direct effects on tumor cells, IFN-λ also modulates immune responses by enhancing DC activation and supporting adaptive immunity, making it a promising candidate for cancer immunotherapy [77]. Noteworthy, IL-29 (IFN-λ1) has emerged as a promising immunotherapeutic cytokine for cervical cancer. In the SiHa cervical cancer cell line, IFN-λ1 inhibits cell proliferation and induces apoptosis via upregulation of the key cell cycle regulators p18 and p27 and the pro-apoptotic receptor TRAILR1. The dual activity of cell cycle inhibition and apoptosis induction by IFN-λ1 highlights its potential as anti-tumor therapeutic agent [105]. Local delivery of IFN-λ near tumors strongly potentiates NK cell-mediated clearance [106].

Aurora kinase inhibitors (AURKi) have emerged as potent activators of innate immune signaling in cancer cells by inducing type I and III IFN responses activating STAT1 signaling and enhancing CD8^+^ T cell infiltration within the TME through endogenous retroviruses (ERVs)-mediated immune activation [107]. This occurs via RIG-I and MAVS signaling and can bypass defective STING pathway in certain tumors [108, 109]. The anti-tumor efficacy of AURKi is dependent on an intact immune system, as evidenced by reduced tumor suppression in immunodeficient mouse models [110]. Moreover, the STING and IFN pathways can act synergistically to enhance anti-tumor immunity, with IFN-λ serving a key role in innate immune priming and tumor surveillance [111, 112].

The accumulating evidence positions IFN-λ as a promising immunotherapeutic agent, particularly when optimized through combinatorial strategies targeting both tumor cells and the TME. This approach not only enhances anti-tumor immunity, but also minimizes adverse effects [113]. However, a deeper understanding of IFN-λ signaling dynamics might be critical for exploiting its therapeutic potential in oncology.

### Role of IFN-λ in virus infections

Next to its role as a biomarker, a potential therapeutic utilization of IFN-λ upon viral infection has been shown. The interplay between innate and adaptive immunity is crucial for determining the outcome of viral infections. Early anti-viral responses, particularly those mediated by IFNs, serve as essential barriers to uncontrolled viral replication and excessive inflammation [114, 115]. Understanding how specific IFN subtypes shape immune dynamics during viral infection has recently provided insights into mechanisms of viral control and disease progression. This has been particularly evident in infections, such as SARS-CoV-2, where timing and the amplitude of IFN responses critically affect disease progression [116, 117].

### IFN-λ in viral infections: mechanism and clinical relevance

The contribution of IFN-λ in the host anti-viral defense system has been extensively studied across various viral infections including those linked to malignant transformation [1, 3, 25, 118]. Using different mouse models of viral infection, IFN-λ effectively protects against DNA viruses, like herpes simplex virus (HSV)2, but shows limited protection against RNA viruses, such as encephalomyocarditis virus (EMCV) and lymphocytic choriomeningitis virus (LCMV) [68]. IFN-λ activates ISGs [119] through the JAK-STAT signaling pathway [31], inhibiting viral replication and spread, promoting degradation of viral RNA, and enhancing antigen presentation thereby acting as a first-line defense against viruses at barrier surfaces [18, 120]. In addition, IFN-λ has been shown to inhibit the replication of hepatitis C virus (HCV) and hepatitis B virus (HBV) indicating its potential as an alternative to IFN-α treatment in resistant HCV patients [121–123]. A link between human papilloma virus (HPV) infection and IFN-λ has been reported with elevated expression levels of IFN-λ1, IFN-λR1 and ISG15 in patients with low risk of HPV infection compared to those with high-risk of HPV infection [124]. Additionally, IFN-λ can inhibit the infection in specific cellular contexts with human immunodeficiency virus type 1 (HIV-1) [125] in macrophages and HSV-1 infection in human astrocytes and neurons [126]. Despite these effects, its anti-viral activity is generally weaker, but more sustained than that of IFN-α reflecting differences in signal transduction patterns and kinetics of gene regulation [32].

IFN-λ is primarily induced in respiratory viruses, such as influenza A virus (IAV) [127–129]. However, these viruses develop mechanisms to diminish its anti-viral activity. For example, IAV and respiratory syncytial virus (RSV) activate the epidermal growth factor receptor (EGFR), which suppresses the IRF1–mediated IFN-λ production thereby facilitating viral replication [130]. Similarly, RSV suppresses IFN-λ production by inhibiting key transcription factors, like IRF-3 and NF-κB [131, 132]. Recent studies indicate that excessive IFN-λ signaling may activate suppressor of cytokine signaling 1 (SOCS1) dampening components of the IFN-λ signaling cascade and downstream ISG responses thereby potentially limiting anti-viral efficacy [17]. These findings highlight that the IFN-λ activity is tightly regulated.

Distinct non-redundant activities of IFN-λ members have also been identified. For example, treatment with IFN-λ3 modulates T cell activation by not only restricting the differentiation of central memory into effector memory T cells, but enhancing the strength and quality of CD8^+^ T cell responses, particularly in vaccine settings [133]. Next to direct anti-viral effects, IFN-λ2 regulates immune cell function including macrophage activation and adaptive immune responses thereby bridging innate and adaptive immunity. IFN-λ1 enhances TLR-mediated B cell activation and supports innate immune priming, while IFN-λ4 is associated with impaired viral clearance, especially in HCV infection [27, 61].

Interestingly, inhibition of all IFN-λs by blocking the IL-28 Rα receptor has been shown to enhance the immune response to influenza vaccines by increasing B cell activation and IgG production [134]. Despite these data, it is unclear how type III IFN signaling regulates B cell responses, as various IFN-λ subtypes have distinct immunoregulatory roles across different models. For example, IFN-λ1 has been shown to enhance TLR-mediated activation of B cells [133, 135], while IFN-λ3 has been reported to boost the production of antigen-specific anti-HIV antibodies [133]. IFN-λ4 has been most studied in the context of HCV infection and its expression has been associated with impaired viral clearance and negatively interferes with immune responses to HCV [136, 137]. Genetic variations in the IFN-λ3 gene are associated with differences in the immune response to infections influencing both spontaneous viral clearance and response to therapy, particularly in hepatitis C virus (HCV) infection [138, 139].

Moreover, genetic variations significantly influence IFN-λ function. Common single nucleotide polymorphisms (SNPs) in IFN-λ3, IFN-λ4 and the IFN-λ receptor α-subunit genes have been associated with varying outcomes of IFN-λ-based therapies for chronic HCV infection [140, 141]. The impact of these SNPs may depend on the stage of infection (acute or chronic) and the potential for viral resistance.

Given IFN-λ’s role in modulating the Th1/Th2 balance, SNPs affecting the IFN-λ signaling could alter this balance during infection promoting Th1 responses or inhibiting B cell proliferation and antibody production [99, 142]. This modulation highlights the complex regulatory roles of IFN-λ and has important implications for the magnitude of the host’s immune response. It could provide the rational of the development of future therapies and vaccines targeting both existing and emerging pathogens as well as utilization of these molecules as adjuvants or in the context of malignancies. However, the predictive value of IFN-λ is not uniform and appears to depend on tumor type and immune composition [80].

### Clinical relevance of IFN-λ in viral infections

Given its role at the interface of innate and adaptive immunity, IFN-λ has attracted attention as a biomarker in both infectious diseases and cancer. The measurement of IFN-λ in patients’ serum could reflect early and ongoing immune activation and serve as a non-invasive indicator of immune response dynamics [143]. Elevated IFN-λ serum levels have been observed in several viral infections, including COVID-19, and often correlate with disease severity suggesting a prognostic value. In oncology, IFN-λ expression signatures within tumors correlate with immune cell infiltration and responsiveness to immunotherapies. Although data on IFN-λ remain limited, studies on other IFNs, such as IFN-γ, have established their clinical relevance as biomarkers implying a comparable potential of IFN-λ in disease monitoring and outcome prediction.

Next to its use as biomarker, a potential therapeutic utilization of IFN-λ upon viral infection has been shown. Santer and co-authors show that administration of pegylated IFN-λ (PEG-IFN-λ) accelerated viral clearance in COVID-19 patients [144] and results in reduced SARS-CoV-2 RNA levels compared with placebo (NCT04354259) [145]. Another randomized, single-blind trial (NCT04331899) involving adults with mild to moderate COVID-19 demonstrated a reduced duration of viral shedding and symptoms upon PEG-IFN-λ treatment. Analysis into SARS-CoV-2–specific T cell and Ab responses between placebo- and IFN-λ–treated participants demonstrated that IFN-λ is activating ISGs in IFN-λR1-expressing peripheral immune cells including plasmacytoid DCs and B cells. This treatment does not impair SARS-CoV-2–specific Ab levels or delayed immune activation of virus-specific T cell responses [146]. Thus, IFN-λ represents a promising early treatment strategy for COVID-19 patients, enhancing early innate anti-viral defenses without impairing peripheral adaptive immunity and exerting systemic inflammation.

Beyond COVID-19, PEG-IFN-λ demonstrates a strong anti-viral efficacy against hepatitis B, C, D and E viruses in both clinical and pre-clinical settings [147–149]. Compared to pegylated IFN-α2a, PEG-IFN-λ induced a faster decline in HBV DNA and surface antigen levels with reduced systemic toxicity, while IFN-α2a achieved higher seroconversion and virologic suppression rates. In some patients pre-treated with entecavir, IFN-λ therapy further enhanced NK cell polyfunctionality [150]. Differences remain in virologic suppression and seroconversion rate depending on treatment regimens. The currently ongoing clinical trials with IFN-λ for the treatment of viral infections are summarized in Table 4.

#### Unexpected role of IFN-λ: possible pro-tumorigenic effects

While IFN-λ is widely recognized for its anti-viral and anti-cancer properties, accumulating evidence indicates that it can also exert pro-tumorigenic effects by promoting tumor immune evasion in various cancers, which is summarized in Table 5. These paradoxical roles raise important questions about the context-dependent actions of IFN-λ in the TME.

**Table 5:** Anti–tumor and pro-tumorigenic features of IFN- λ

anti-tumor effects	pro-tumorigenic effects
recruitment of NK and CD8^+^ T cells [115]	reduced recruitment of effector cells promotion of immune exhaustion [151]
inhibition of proliferation [32]	reduction of cytotoxic T cell activity [80]
increased antigen presentation [152]	increased IL-10, TGF-β secretion [153]
induction of apoptosis [154]	alteration of macrophage polarization [79]
improved immunotherapy response induction of cell cycle arrest [154]	upregulation of immune checkpoint molecules [155]
activation of ISGs and anti-viral pathways in tumor cells [156]	facilitation of immune evasion chronic signaling leading to disinvitation [80]
promotion of Th1 responses [75]	suppression of Th2 balance [75]

One key mechanism mediating these effects involves the suppression of anti-tumor immune responses by negatively influencing the recruitment and activation of cytotoxic T cells [157, 158] or NK cells [159, 160] into the TME, endogenous IFN-λ can facilitate tumor evasion and progression. Furthermore, cancer-specific expression of IFN-λ can dysregulate multiple downstream-related pathways, including cytokine-cytokine receptor interactions, humoral immune responses and JAK-STAT signaling pathways, which may be drivers of cancer development. Mechanistically, IFN-λ can downregulate the expression of critical pro-inflammatory chemokines and cytokines, such as TNF-α and IL-12, which are essential for T cell migration and activation, thereby diminishing the efficacy of adaptive immune responses [161]. Clinical observations support the findings: In advanced melanoma, elevated levels of IFN-λ correlated with a reduced CD8^+^ T cell infiltration leading to a poorer patients’ prognosis [162]. Similarly, increased IFN-λ expression correlates with decreased numbers of T cells, myeloid conventional DCs as well as an increased prevalence of exhausted CD4 + T cells within the TME [163]. IFN-λ-mediated induction of T cell exhaustion is characterized by diminished effector functions and upregulation of inhibitory receptors, such as PD-1 [163]. Concerning NK cell function, IFN-λ can alter the expression of activating and inhibitory receptors, consequently diminishing their cytotoxicity against tumor cells. Additionally, it promotes the secretion of immunosuppressive cytokines, such as IL-10 [102] The shift of macrophage polarization from the pro-inflammatory M1 phenotype towards immune suppression is another important aspect of IFN-λs role in tumor progression and immune evasion due to secretion of immune suppressive IL-10 and TGF-$\beta$ [79, 164, 165]. It should be also noted that IFN-λ also contributes to a pro-inflammatory milieu through the induction of IL-6 secretion that paradoxically promotes tumor cell proliferation [166] the complexity of IFN-λs action, while simultaneously support immune responses, but fostering conditions that promote tumor control.

At the genetic and epigenetic level, gene copy numbers of IFN-λ2 and IFN-λ3 variants are increased in multiple cancer types compared to healthy individuals. These alterations are often associated with an elevated number of mutations in the TP53 gene [45] lower levels of DNA methylation may contribute oncogene activation and tumor growth as shown in CRC and pancreatic cancer. Such changes have been associated with cancer progression and immune evasion [45], leading to T cell dysfunction and a sustained inflammatory environment.

### Limitations and future directions

**Fig. 3:**
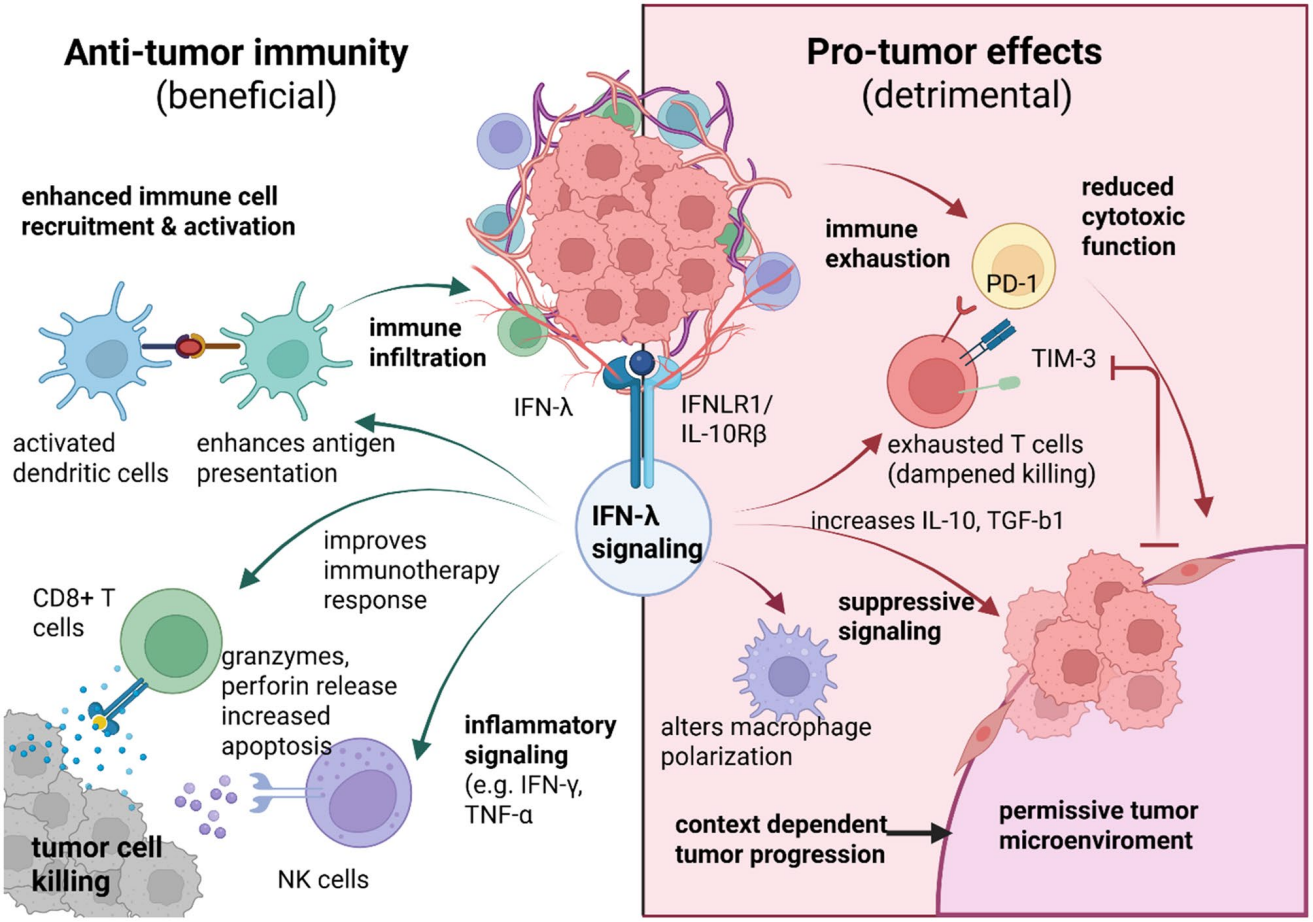
Dual role of IFN-λ in cancer. Schematic overview of the context-dependent effects of IFN-λ in the tumor microenvironment. IFN-λ promotes anti-tumor immunity by enhancing immune cell recruitment and activation, but may also induce immune exhaustion, suppress cytotoxic responses, and promote tumor progression under specific conditions

The dual roles of IFN-λ in immune modulation highlight its complex nature as both a therapeutic agent and a potential facilitator of tumorigenesis (Fig. 3).

While it holds promise in the treatment of viral infections and cancer, its context-dependent effects necessitate a deeper understanding of its specific mechanisms of action and challenges the traditional classification of IFNs as protective cytokines. Instead, IFN-λ should be considered a regulator of immune balance, capable of both promoting host defence and contributing to immune dysfunction. This duality is particularly evident in cancer, where the same signalling pathways that enhance immune surveillance may, under chronic or dysregulated conditions, drive immune exhaustion and tumor progression.

A key unresolved question is how the spatial restriction of IFN-λ signalling influences systemic immunity. While its localized activity at epithelial barriers provides clear advantages in limiting inflammation, it may also constrain the development of robust systemic immune responses. This has important implications for both anti-viral immunity and cancer immunotherapy, where effective systemic responses are often required. Another critical aspect is the temporal dynamics of IFN-λ signalling. Early and transient activation appears to be beneficial in controlling viral infections and initiating anti-tumor immunity, whereas prolonged exposure may lead to immunosuppressive effects. Understanding these dynamics will be essential for optimizing therapeutic interventions, particularly in the context of chronic infections and cancer. Furthermore, the interplay between IFN-λ and other immune pathways, including type I IFNs, immune checkpoints, and inflammatory signalling cascades, remains incompletely understood. These interactions likely determine the overall outcome of IFN-λ signalling and represent important targets for combination therapies. For example, integrating IFN-λ with immune checkpoint blockade may enhance anti-tumor responses, while mitigating immune exhaustion.

From the translational perspective, further investigations and clinical trials have to be performed to fully understand how IFN-λ modulates immune responses and tumor biology and how genetic variations and expression levels of IFN-λ in different cancers can be harnessed effectively in therapeutic strategies, while mitigating its potential of tumor evasion and progression. Biomarkers such as IFN-λ receptor expression, genetic polymorphisms, and immune signatures within the TME may help identify patients most likely to benefit from treatment. Therefore, in depth analysis of IFN-λs role in the tumor immune landscape as well as careful consideration of dosing, timing, and combination strategies will be critical for the development of targeted therapies that maximize its benefits, while minimizing its associated risks.

## Conclusion

IFN-λ represents a unique and versatile component of the immune system with significant translational potential. Its ability to mediate localized anti-viral and immunomodulatory effects, while limiting systemic inflammation distinguishes it from other IFN families. However, in cancer, IFN-λ exhibits context-dependent dual functions, contributing to both anti-tumor immunity and under certain conditions immunosuppression, highlighting both opportunities and challenges for clinical application. A deeper mechanistic understanding, coupled with biomarker-driven patient selection and rational combination therapies (e.g., with checkpoint inhibitors or anti-virals), will be essential to fully harness the therapeutic potential of IFN-λ in infectious diseases and oncology. Future studies should also explore its role in shaping tumor and immune microenvironments, optimizing cloning regimens, and defining disease-specific strategies to maximize efficacy, while minimizing adverse effects.

## Data Availability

Not applicable.

## Abbreviation

Ab: Antibody
APC: Antigen-presenting cell
DC: Dendritic cell
EMCV: Encephalomyocarditis virus
GAS: Gamma-activated sequence
HBV: Hepatitis B virus
HCV: Hepatitis C virus
HIV-1: Immunodeficiency virus type 1
HPV: Human papilloma virus
IAV: Influenza A virus
IFN: Interferon
IFNL: Interferon lambda
IFNR: Interferon receptor
IRF: Interferon regulated factor
ISG: Interferon-stimulated gene
ISRE: Interferon-stimulated response element
JAK: Janus kinase
LCMV: lymphocytic choriomeningitis virus
MAPK: Mitogen-activated protein kinase
NSCLC: Non-small cell lung carcinoma
PD1: Programmed cell death 1
PD-L1: Programmed death ligand 1
pDC: Plasmacytoid dendritic cell
PEG: Pegylated
RSV: Respiratory syncytial virus
SNP: Single nucleotide polymorphism
STAT: Signal transducer and activator of transcription
TLR: Toll-like receptor
TME: Tumor microenvironment
Treg: Regulatory T cell
TYK2: Tyrosine kinase 2
